# Iodine-125 induces apoptosis via regulating p53, microvessel density, and vascular endothelial growth factor in colorectal cancer

**DOI:** 10.1186/1477-7819-12-222

**Published:** 2014-07-17

**Authors:** Zhenhuan Ma, Yong Yang, Guokai Yang, Jia Wan, Guojian Li, Ping Lu, Lingjuan Du

**Affiliations:** 1 Department of General Surgery, the Second People’s Hospital of Yunnan, Kunming 650021, China; 2 Center of Vascular Surgery, The Fourth Affiliated Hospital, Kunming Medical College, Kunming 650021, China

**Keywords:** Colorectal cancer, HCT-8 tumor model, I-125 interstitial brachytherapy, Microvessel density, p53 protein, Vascular endothelial growth factor

## Abstract

**Background:**

Iodine interstitial brachytherapy has been widely reported for treating colorectal cancer (CRC). However, the inhibitory molecular mechanism of iodine-125 (I-125) on CRC has not been reported.

**Methods:**

To illustrate the inhibitory mechanism of iodine-125 (I-125) on CRC, we established the animal models of CRC via the injection of HCT-8 cells into nude mice. Subsequently, the I-125 granules were implanted into the tumor of the animal model at different dosages. Proliferating cell nuclear antigen and terminal transferase dUTP nick end labeling were used to detect the apoptosis of the tumor cells. Immunohistochemistry SP staining was used to measure the expression of p53 protein. The protein levels were examined with western blot and ELISA. Meanwhile, microvessel density (MVD) was counted by endothelial cells immunostained by anti-CD34 antibody.

**Results:**

The results showed that I-125 protests against CRC via increasing the protein level of p53 and decreasing the level of vascular endothelial growth factor (VEGF), leading to the decrease of MVD in CRC (*P* <0.0001). An effective inhibition dosage of I-125 ranged from 0.4 to 0.8 mCi.

**Conclusions:**

The inhibitory mechanisms of iodine on CRC acted through an increase in the level of p53 and a decrease in the level of VEGF, resulting in a decrease of MVD.

## Background

According to a previous report [1], colorectal cancer (CRC) is the third most common cancer in men and the second in women worldwide, with most cases occurring in developed regions. Existing treatments are ineffective in the cure of CRC [2]. Further, the cancer’s size and/or position may make surgery difficult and thus patients survive for only a short time after surgery [3]. Additionally, gastrointestinal stromal tumors (GISTs), a subset of soft tissue sarcomas, have proven to be insensitive to chemotherapy and radiotherapy [4].

Radioactive iodine-125 (I-125) seed implantation can improve the target volume dose with a high level of radioactive iodine. The tumor, which is refractory and insensitive to chemotherapy and radiotherapy, can be effectively controlled via iodine treatment [5]. Furthermore, the preservation of urinary, sexual, and rectal quality of life is excellent at long-term follow-up for the patients implanted with I-125 [6]. Iodine interstitial brachytherapy has been widely reported for treating CRC. Segmental intraluminal instillation of iodine was advocated to prevent anastomotic recurrence after colonic resection for CRC [7]. Most patients with recurrent or metastatic CRC underwent radio-immune-guided surgery [8]. I-125 has also been widely applied to treat other cancers. For instance, I-125 brachytherapy implantation is a safe and effective treatment strategy for patients with both lower urinary tract symptoms and localized prostate cancer [9]. Combined techniques with I-125 implant show better anti-tumor effects. Further, the addition of external beam radiotherapy can confer a significant biochemical control for prostate cancer when it is added to I-125 brachytherapy [10]. The patients with IS-IV (interval survival for stage IV pancreatic cancer), received radical treatment, are significantly longer than those who received palliative treatment. Multiple treatments play an important role in improving the IS-IV of patients who received radical treatment [11].

The inhibitory molecular mechanisms of iodine on some cancers have been reported [12-14]. However, the inhibitory molecular mechanism of I-125 on CRC is still widely unknown. Understanding the molecular mechanism will be beneficial to make full use of the I-125 brachytherapy implant techniques effectively and safely. CRC models with nude mice are readily available [15,16], allowing for the exploration of the inhibitory molecular mechanisms of I-125 on CRC. We established a CRC animal model using nude mice, followed by the implant of the I-125 granules at different dosages. The inhibitory molecular mechanisms were explored and are described herein.

## Methods

### Cell culture

As previously described [17], HCT-8 cells were maintained under serum-free conditions using McCoy’s 5A medium supplemented with 4 μg/mL of transferrin, 5 μg/mL of insulin, and 10 ng/mL of vascular endothelial growth factor (VEGF) in a 5% CO_2_ at 37°C and 100% humidity, and passaged every 2 or 3 days.

### Orthotopic implantation

All the protocols were approved by the animal care and ethics committee of the Second People’s Hospital of Yunnan (Kunming, China). Informed consent was obtained from each patient before the study. BALB/c nude male mice were raised in a HEPA-filtered environment. HCT-8 labeled cells were subcutaneously injected into the mice. At 1 cm^3^, the xenograft was excised and minced for implantation into other 4- to 6-week-old BALB/c nude mice. One 1 mm^3^ piece per mouse was subcutaneously implanted to the axillary region of 50 other BALB/c nude male mice, as previously described [18].

### I-125 brachytherapy seed implant

Five days after the tumors were implanted, an I-125 seed or an inactive sham seed was inserted into the tumor areas of the mice model. Every 10 mice received experimental seeds with the radio dosage of 0.2, 0.4, and 0.8 mCi. Ten control mice received the sham seeds or did not receive the implant. Every 5 days, the tumor volume was calculated using the modified ellipsoid formula 1/2 (Length × Width^2^). All mice were sacrificed after 15 days.

### The determination of p53

Immunohistochemical SP staining was used to determine the protein levels of p53 according to a previous report [19]. Briefly, 5-μm formalin-fixed paraffin-embedded sections from the tumors were boiled for 5 min in the presence of lead thiocyanate solution. Sections were incubated at room temperature for 30 min with a 1:1,000 dilution of the polyclonal antibody, CM-1 (Covance, Inc., Beijing, China) against p53 protein after blocking endogenous peroxidase activity with goat serum. Immunohistochemical staining was performed using the streptavidin-biotin complex procedure according to the manufacturer’s direction (Advanced Technology & Industrial Co., Ltd., Hong Kong). As a control, the same procedure was carried out except that the primary antibody was replaced by PBS. The neoplastic areas were examined for immunohistochemical staining by one investigator, blinded to the p53 mutation status. Only the nuclear immunostaining was considered as positive for p53 protein accumulation. The level of protein accumulation was scored as 0 (no detectable immunostaining), 1 (few nuclei), 2 (up to 10% nuclei), 3 (10 to 50% nuclei), and 4 (>50% nuclei). The numerical scoring was confirmed by a second independent examination, blinded to the initial score.

### Proliferating cell nuclear antigen (PCNA) and terminal transferase dUTP nick end labeling (TUNEL) assay for apoptosis

PCNA was examined to investigate cell proliferation, which can reflect the degree of the cellular apoptosis. All cells were fixed in PBS formalin, embedded in paraffin and stained. PCNA was performed using a labeled streptavidin-biotin. Anti-PCNA monoclonal antibody (DAKO, Carpenteria, CA, USA) reacted exclusively with nuclei.

TUNEL was also performed to examine the apoptosis according to a previous report [20]. All cells were suspended in PBS buffer by gently vortexing the vials and aliquoted to approximately 1 × 10^6^ cells/mL per test. The cells were collected via centrifuge for 5 min (300 *g*) and resuspended in 1 mL of the wash buffer in each tube. After washing three times, 50 μL of the TdT enzyme mixture were added to the sample and incubated for 60 min at 37°C in a water bath. Subsequently, the cells were washed three times and resuspended in 0.1 mL of the antibody labeling mix. The tubes were incubated in the dark for 30 min at room temperature. The cells were washed three times and suspended in 0.9 mL PBS. One hundred μL propidium iodide/RNase were added to each tube. After 3 hours of staining, the samples were analyzed by flow cytometry.

### Detection for the expression of vascular endothelial growth factor (VEGF) and quantitation of microvessel density (MVD)

The expression level of VEGF was determined according to Takahashi standard [21]. Immunohistochemical analyses, using the antibodies against VEGF and PCNA, were performed in 50 human colon carcinomas. Vessels were quantitated by light microscopy and the intensity of staining for VEGF was assessed on a scale of 0 to 3+. Proliferation was determined by counting the number of PCNA-positive cells per 500 tumor cells. The high expression of VEGF was correlated with the extent of neovascularization and the degree of cellular proliferation.

MVD, a measure of angiogenesis in tumors, has been shown to be a prognostic indicator that correlates with an increased risk of metastasis in various cancers [22]. MVD was measured according to a previous report [23].

### Western blotting analysis

After I-125 treatments with different dosages, cells were washed in Dulbecco’s phosphate-buffered saline (DPBS) (Life Technologies, Grand Island, USA) and incubated in cysteine-free/methionine-free DMEM (Life Technologies, Inc.) for 30 min. One 6-cm plate containing 1 × 10^6^ cells was used for per sample. Cell lysates were prepared as previously described [24]. Total protein in lysates was quantified using Bradford reagent (Bio-Rad Laboratories, Inc., Richmond, CA, USA). All proteins were separated on 10% SDS-PAGE. Western blot analysis was conducted with the monoclonal antibody against p53 (clone BP53-12 at a 1:50 dilution, Novocastra, Newcastle upon Tyne, UK) and polyclonal antibody against VEGF (clone A-20 at a 1:300 dilution, Santa Cruz Biotechnology, Santa Cruz, CA, USA). β-actin (Sigma-Aldrich, St. Louis, MO, USA) was used as a loading control in the western blot.

### ELISA

The concentration of p53 in the treated and untreated tissues was determined using p53 Abs ELISA kit [25] (Medical & Biological Laboratories Co. Ltd., Nagoya, Japan). The concentration of VEGF in the treated and untreated tissues was determined using an ELISA kit with the antibodies which recognize VEGF165 [26] (R&D Systems Inc., Minneapolis, USA). Glyceraldehyde-3-phosphate dehydrogenase hybridization was used as a loading control.

### Statistical analysis

The statistic differences between two means were compared by one-way analysis of variance (ANOVA). Fisher’s least significant difference test was used as post hoc tests of significant differences between two parameters. Data was analyzed using StatView 5.0 software (Abacus systems, Berkley, CA, USA) with a *P* value of <0.05 accepted as significant.

## Results

### The effects of I-125 implant on tumor volume

**Figure 1:**
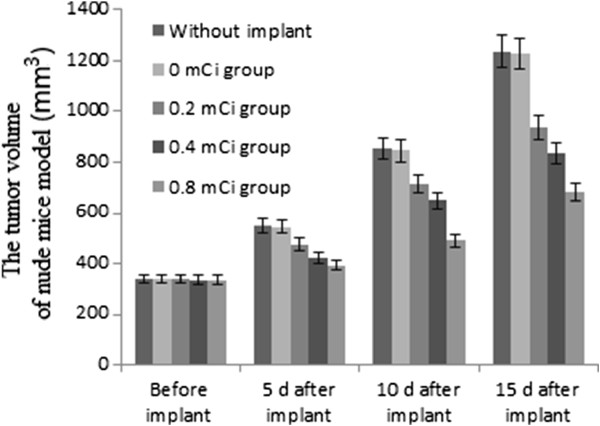
**The tumor volume of nude mice model before/after I-125 seed implant.** Comparing with the control group, *P <0.001 using least significant difference method. Each bar represented the mean ± SD of three independent experiments.

Before the I-125 seed implant, the tumor volumes were almost the same. After the seed implant, the tumor volume in the 0.8 mCi group was reduced by up to 40% compared with those from the controls with 0 mCi (Figure 1); the difference was significant (*P* <0.001). The effective inhibition dosage of I-125 ranged from 0.4 to 0.8 mCi (Figure 1).

### Inhibitory rate of different dosages

The weight of the different tumors was 5.26 ± 0.31, 5.27 ± 0.25, 4.13 ± 0.13, 3.47 ± 0.17, and 2.83 ± 0.16 g in blank, 0, 0.2, 0.4, and 0.8 mCi groups, respectively, giving a respective growth inhibition of 0, 21.5%, 34.0%, and 46.2%. Thus, the 0.8 mCi I-125 dosage showed effective inhibitory results for CRC.

### The assay for apoptosis

**Figure 2:**
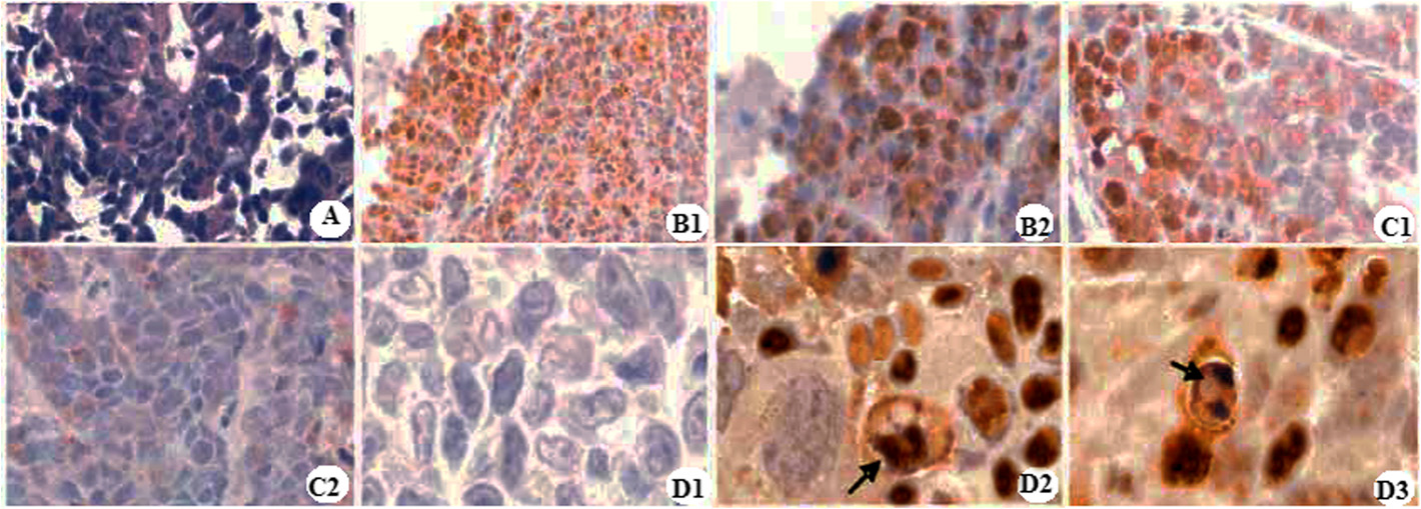
**HCT-8 cell transplanted tumor (HE** × **400). (A)** Strong positive expression of PCNA protein on different phases after I-125 seed implantation; **(B1)** HCT-8 cell transplanted tumor before day 5 in the 0 mCi group (SP × 200); **(B2)** Day 5 in the 0.8 mCi group (SP × 400); **(C1)** Day 15 in the 0 mCi group (SP × 400); **(C2)** Day 15 in the 0.8 mCi group (SP × 400); **(D1)** 0 mCi group, a few apoptosis cells were seen, nuclei were stained blue and small nucleoli were seen; **(D2)** 0.8 mCi group, characteristic findings of cell apoptosis, germination was observed on day 10; **(D3)** 0.8 mCi group, vacuolization in the cytoplasm occurred in the apoptotic cell on day 15.

The apoptosis was visualized in the tumors treated with I-125 under an oil microscope. According to a previous report, brown colored positive apoptotic cells were observed and normal cells were in a blue color with the TUNEL method [27]. Figure 2A shows that the nuclei were colored blue in the normal cancer cell. Five days after the I-125 seed implantation, the nuclei were stained a brown color with decreased expression of PCNA in apoptosis cells while the nuclei were of a normal color in the 0 mCi group (Figure 2B1 and B2). Fifteen days after I-125 seed implantation, the nuclei disappeared because of the emerging destructive stage of apoptosis, while a few cells showed apoptosis in the 0 mCi group (Figure 2C1 and C2). The TUNEL assay showed normal colon cells with a blue color while the germination of cells was found in the 0.8 mCi group (Figure 2D1 and D2). Vacuolization in the cytoplasm occurred in the apoptosis cell on day 15 of I-125 implantation (Figure 2D3).

### The association between MVD and VEGF

The MVD for the five groups was 50.19 ± 21.38, 51.30 ± 20.26, 41.67 ± 17.56, 32.50 ± 10.95, and 22.62 ± 7.14, respectively. The expression of VEGF and MVD was closely related with the development of CRC. The results suggested that high protein level of VEGF caused high levels of MVD, which would increase the risk of colon cancer.

### The association between weights and MVD-VEGF

We guessed that the weight of model mice might be affected by the levels of MVD and VEGF. Thus, the association between the weight and levels of MVD-VEGF was investigated here. The Spearman’s rank correlation coefficient for the association between the weigh and the levels of MCD or VEGF in the tumors was 0.85 and 0.72, respectively. Both *P* values were less than 0.01. The results suggested that the weight was strongly related with the levels of MVD or VEGF.

### The relative protein levels of p53 and VEGF

**Figure 3:**
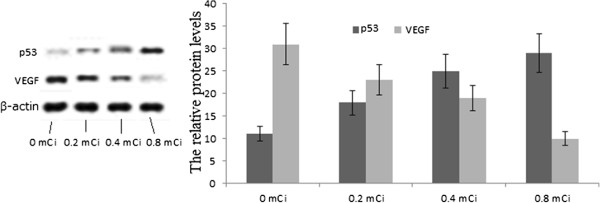
**Western blotting analyses for the relative protein levels of p53 and VEGF in 0, 0.2, 0.4, and 0.8 mCi I-125-treated groups.** β-actin was used as a loading control. Each bar represented the mean ± SD of three independent experiments.

Compared with the control group, the expression level of p53 was up-regulated when it was treated by I-125 seeds from low- to high-dosage (Figure 3), suggesting that I-125 can increase the expression level of p53. p53 is an important cancer repressor [28], and thus the mechanism of iodine inhibition of CRC growth acts through activation of the levels of p53. Inversely, comparing the control group, the expression of VEGF was down-regulated when it was treated by I-125 from low- to high-dosage (Figure 3), suggesting that I-125 can reduce the expression of VEGF. Anti-VEGF has been studies in clinical trials for cancer therapy [29]; thus, iodine treatment is a better method for inhibiting the growth of CRC via the activation of anti-VEGF.

### The concentration of p53 and VEGF

**Figure 4:**
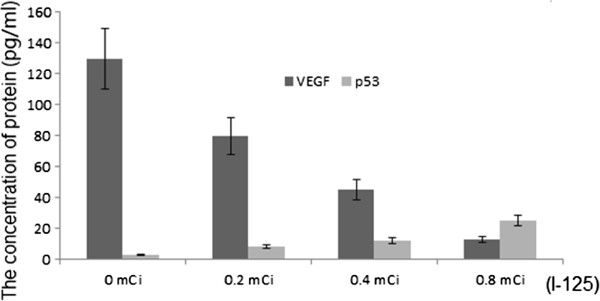
**The concentration of p53 and VEGF in 0, 0.2, 0.4, and 0.8 mCi I-125 treated groups determined by ELISA.** Each bar represented the mean ± SD of three independent experiments.

Compared with the control group, the concentration of p53 reached the highest level when the rats were treated with I-125 at the highest dosage (Figure 4), suggesting that I-125 can increase the concentration of p53. Just as analyzed by western blotting, p53 was an important cancer repressor, and the mechanism for iodine inhibiting the growth of CRC was to increase the level of p53. Inversely, compared with the control group, the concentration of VEGF was reduced to the lowest level when it was treated by I-125 at the highest dosage (Figure 4), suggesting that I-125 can reduce the concentration of VEGF.

## Discussion

Cancer is a leading cause of death in the world and I-125 radiation therapy has proved effective in the treatment of various carcinomas [30-34]. The theoretical benefit of seed permanent implantation as a salvage treatment is to enhance disease control in the region of recurrence and to minimize the injury to the surrounding tissues [35,36]. However, the molecular mechanisms for I-125 inhibiting CRC are widely unknown. We were therefore interested to explore said mechanisms. Herein, we gained significant results in using I-125 seed implantation to treat CRC mice models. We firstly reported the molecular mechanisms involved in the inhibition of CRC by I-125, mainly showing that a suitable dosage of I-125 can increase the expression of p53, which inhibits the expression of VEGF and causes lower MVD, leading to the cell apoptosis. The findings also indicated that MVD and VEGF were reliable combined predictors of prognosis in CRC. On the other hand, these findings support the hypothesis that VEGF is an important angiogenic factor in primary and metastatic human CRC. VEGF expression and vessel counts might aid in predicting patients at risk for metastasis from CRC, which is consistent with previous reports [37,38].

Regarding the tumor apoptosis caused by I-125 treatment, one important issue remains uncertain, namely that Figure 2D2, D3 is not completely clear and thus apoptosis may be confounded with mitotic death and pre-micronuclei formation. However, apoptosis can be evaluated by peroxidase-TUNEL (brown) counterstained with hematoxylin (blue) [39]. Thus, we still thought I-125 treatment caused the apoptosis of CRC but not mitotic death and pre-micronuclei formation since the nuclei were a brown color.

Epidemiologic studies have consistently reported positive associations between the weight and CRC risk for adults [40-42]. Achieving optimum BMI levels and weight reduction in the population appears to offer the greatest health benefits and decrease the risk of CRC [43,44]. Herein, we found that the I-125 treatment resulted in mice weight loss, which was the main reason for I-125 controlling the development of CRC. Thus, the molecular mechanisms for I-125 inhibiting CRC were that I-125 treatment increases the expression of p53 and reduces the expression of VEGF, which leads to a decrease of MVD. All the parameters were closely related with the weights of the mice models. The information should be beneficial to improve the treatment of various cancers with iodine.

The efficacy of iodine in treating various cancers is obvious [45-47], but its adverse effects are also protruding [48,49]. Even though the risk of I-125 for persistent side effects is rather small, these data do emphasize the need to carefully select the patients for I-125 treatment. The CRC patients who are thought to be at moderate to high risk for recurrence, should use the minimally effective dose of I-125 activity, in an attempt to maximize the potential benefit while minimizing the risk for adverse events. Here, we found that the effective inhibition dosage of I-125 ranged from 0.4 to 0.8 mCi. The results offer important information for subsequent clinical trials and will be beneficial to utilize the iodine therapy techniques effectively and safely. Considering the side effects of I-125, controlled trials with a larger sample size and longer follow-up are recommended in the future.

## Conclusions

In summary, we established animal models of CRC via the injection of HCT-8 cells into nude mice. Subsequently, the I-125 granules were implanted into the tumor of the animal model at different dosages. PCNA and TUNEL were used to detect apoptosis of the tumor cells. I-125 protests against CRC via increasing the protein level of p53 and decreasing the level of VEGF, which leads to the decrease of MVD in CRC. An effective inhibition dosage of I-125 ranged from 0.4 to 0.8 mCi.

## Abbreviations

CRC: Colorectal cancer; I-125: Iodine-125; MVD: Microvessel density; PCNA: Proliferating cell nuclear antigen; TUNEL: Terminal transferase dUTP nick end labeling; VEGF: Vascular endothelial growth factor.

## Competing interests

The authors declare that they have no competing interests.

## Authors’ contributions

LB and ZL played equally important roles in the development of the experimental protocol. L-KY, S-QM, and M-ZH interpreted the results. The corresponding author, YY, was responsible for the tasks of co-ordination arrangements. All authors read and approved the final manuscript.
